# Hot-Air Furnaces

**Published:** 1857-12

**Authors:** 


### HOT-AIR FURNACES

OUGHT not to be tolerated; they ruin the wood-work of any building, ruin the furniture, and, more than all, impair the health of every person who breathes the atmosphere of houses thus heated by them. Warm air relaxes, debilitates, the world over; cool air braces up, gives tone, vigor, power, to the whole frame.

Warm air evaporates every article that has moisture in it, fluids, meats, vegetables—everything; these particles are distributed all through the air of the house, to the exclusion, to that extent, of the life-giving oxygen; so that not one single breath of pure air is taken into the lungs, as long as the person occupies such a house; and when it is remembered that during the most inclement season of the year, there are days, even weeks, during which the very young and the very old of the family, as also the invalids, do not pass outside the door, it is not to be wondered at that there is not one day, during all the winter, in which health dwells in any household so warmed. But a great deal of the ill effect of furnace-heated rooms may be obviated, if the fire-place is always kept open; but in very cold weather, there should be a fire in the fire-place, in order to create a more decided draught towards it, so as to promote a circulation, and carry the bad air more rapidly up through the chimney, and out of the building.

It is a great mistake, and an almost universal one, that sudden changes from one temperature to another are prejudicial to health. If persons will close their mouth, and send all the air to the lungs through the circuit of the head, and thus temper it to the air of the lungs, a positive benefit will result, although there may be a change of forty degrees in a second of time; only one precaution is needed. *Shut your mouth, and keep moving.*

The proof of all this is, railroad conductors are healthy men, as a class, and yet their changes are fifty degrees, hundreds of times in a day.

In addition, it is known to all persons of observation, that the inhabitants of the equable and moderate climates are not long-lived. The "Italian skies," and the "South of France,"

so much boasted of, do not give length of days to those who enjoy their balmy atmosphere.

Our grandsires lived in cozy parlors, and fire-place heated dining-rooms, with passages and halls as cold as Greenland, and yet they boast a higher health than their degenerate sons and daughters. These are facts, and they ought to have a rational consideration. Down, we say, with every hot-air furnace in the land!

